# Assessment of water, sanitation, and hygiene practices and associated factors in a Buruli ulcer endemic district in Benin (West Africa)

**DOI:** 10.1186/s12889-015-2154-y

**Published:** 2015-08-19

**Authors:** Roch Christian Johnson, Gratien Boni, Yves Barogui, Ghislain Emmanuel Sopoh, Macaire Houndonougbo, Esai Anagonou, Didier Agossadou, Gabriel Diez, Michel Boko

**Affiliations:** Laboratory of Hygiene, Sanitation,Toxicology and Environmental Health, Interfaculty Center of Training and Research in Environment for the Sustainable Development, University of Abomey-Calavi (UAC), 01, PO Box 1463, Cotonou, Benin; National Buruli ulcer Control Program; Ministry of Health, Cotonou, Bénin; Anesvad Foundation, General Concha, 28 - 1°, 48010 Bilbao, Spain

## Abstract

**Background:**

Control of neglected tropical diseases (NTDs) requires multiple strategic approaches including water, sanitation and hygiene services (WASH). Buruli ulcer (BU), one of the 17 NTDs, remains a public health issue in Benin particularly in the district of Lalo. The availability of water as well as good hygiene are important for the management of Buruli ulcer particularly in the area of wound care one of the main component of the treatment of BU lesions. Given the growing importance of WASH in controlling NTDs and in order to assess the baseline for future cross-cutting interventions, we report here on the first study evaluating the level of WASH and associated factors in Lalo, one of the most BU-endemic districts in Benin.

**Method:**

A cross-sectional study was carried to assess WASH practices and associated factors in the district of Lalo. Data were collected from 600 heads of household using structured pretested questionnaire and observations triangulated with qualitative information obtained from in-depth interviews of patients, care-givers and community members. Univariate and multivariate analysis were carried to determine the relationships between the potential associated factors and the sanitation as well as hygiene status.

**Results:**

BU is an important conditions in the district of Lalo with 917 new cases detected from 2006 to 2012. More than 49 % of the household surveyed used unimproved water sources for their daily needs. Only 8.7 % of the investigated household had improved sanitation facilities at home and 9.7 % had improved hygiene behavior. The type of housing as an indicator of the socioeconomic status, the permanent availability of soap and improved hygiene practices were identified as the main factors positively associated with improved sanitation status.

**Conclusions:**

In the district of Lalo in Benin, one of the most endemic for BU, the WASH indicators are very low. This study provides baseline informations for future cross-cutting interventions in this district.

## Background

Neglected tropical diseases (NTDs) include 17 tropical diseases that are prevalent in Africa, Asia and South America. They mostly affect the poor rural populations, especially in areas with low coverage of hygiene and sanitation. These diseases are important causes of morbidity, and sometimes generate significant disabilities as well as stigmatization of affected populations, perpetuating poverty [1]. Control of NTDs requires multiple strategic approaches such as preventive chemotherapy; intensive case management; surgery and chronic care; transmission control; information, education and communication and water and sanitation [1]. Indeed, sustainable water, sanitation, and hygiene (WASH) services are essential for the prevention, long-term control, and even elimination of five of the NTDs: soil-transmitted helminthiasis, trachoma, schistosomiasis, lymphatic filariasis (LF), and Guinea worm disease [2]. Reducing levels of these WASH-preventable NTDs not only improves health and alleviates suffering, but can also lead to improved educational outcomes for children and increased economic progress for communities and nations. The WASH sector can significantly affect health and development by targeting WASH activities where NTDs occur and by incorporating behavioral change messages relevant to specific NTDs into existing hygiene promotion efforts [1].

Buruli ulcer (BU), one of the 17 NTDs, is caused by a germ, *Mycobacterium ulcerans*, that mainly affects the skin but that can also affect the bones. It has been reported in over 30 countries. Most of those affected are children under 15 years of age who live in poor, rural communities. Late diagnosis can result in long and costly hospitalizations with significant morbidity and disability [3]. The disease is endemic in eight departments of southern Benin, with among one of the highest levels seen in the district of Lalo [4]. Whether the relationship between the WASH is clearly demonstrated for the five NTDs cited above [2], the link between WASH and BU is still little studied and the results of different studies on a possible association between BU and WASH need further investigations [5]. There is strong evidence that the endemic foci of BU almost always organized around an aquatic ecosystem [3]. However, the WASH sector is vital for the management of BU: BU patients need water for wound care, scar management after skin grafts, taking medications, and for good personal hygiene to prevent complications and secondary infections. Washing the skin lesions with clean water and soap could be protective for those exposed to BU [6]. Indeed, studies on wound care (an important component of the management of BU) have shown that washing wounds daily with clean water and soap is effective in reducing the risk of contamination by various microorganisms [7]. In addition to the above mentioned observations, Buruli ulcer control strategies as well as the community-led total sanitation (CLTS) strategy recommended for WASH in rural settings require significant community mobilization. Community based activities, as a cross-cutting interventions, can therefore be implemented alongside these for efficient use of resources in BU endemic communities where WASH services are poor. Given the growing importance of WASH in controlling NTDs [1], we report here on the first study in Benin to evaluate the level of WASH and associated factors in one of the most BU-endemic communes, to assess the baseline for future community-based cross-cutting interventions.

## Method

### Study site

**Fig. 1:**
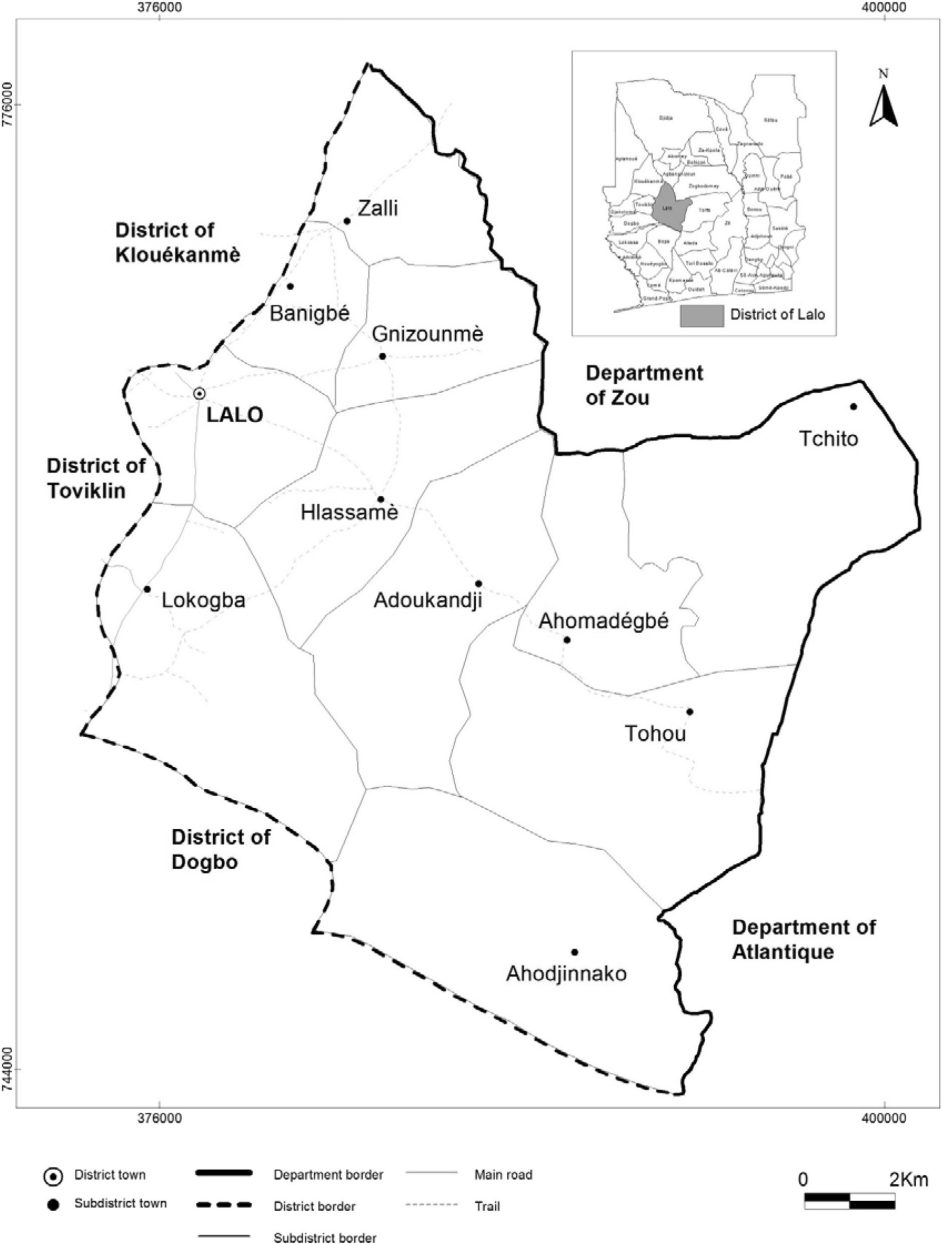
Map of the commune of Lalo

This study was conducted in the district of Lalo, one of the administrative divisions of the Couffo department in Benin. Covering an area of 432 square kilometers, the district of Lalo is divided into 11 sub-districts, with 56 villages and 5 urban neighborhoods, and an estimated population of 119,080 in 2014 (Fig. 1).

### Study design

A cross sectional study was conducted from July to December 2013 using a pretested and structured questionnaire completed by qualitative informations obtained from in-depth interviews of patients, care-givers and community members.

### Sampling

Within the framework of this study (30) clusters of twenty (20) heads of households were selected in all administrative subdivisions of the commune. The number of cluster per subdistrict depends on the population of each subdistrict. Six hundred (600) heads of households were thereby selected across the 11 sub-districts of the district and interviewed using a questionnaire designed for this purpose. After the development of the questionnaire and other data collection tools by the research team, the validation of the questionnaire was made in several steps. A pre test was conducted in the commune of Lalo. Subsequent corrections and rephrasing were made. The questionnaire and pre-tested tools were then submitted to the ethical review committee and the comments of this committee were taken into account in the final version.

For the qualitative component of this study, 15 participants (one per sub-district, two care-givers and two patients) were selected and related issues were discussed. An experienced social scientist moderated all the in-depth interviews. In addition to handwritten notes during the interview, interviews were tape-recorded and later transcribed and translated into French. The main issues addressed by the in-depth interviews were those affecting the water, sanitation and hygiene status. Privacy and confidentiality of the interviewees, as well as good interaction between individuals and interviewer, was maintained during the data collection and interview time.

### Operational definitions

Based on the WHO/UNICEF 2013 report on WASH [8], an unimproved (poor) water source is water from a dam or pool, or stagnant water from a river, stream or rainwater tank. Improved (good) water sources are water piped into the residence, from a human-powered drill or from a water tower. Households with unimproved (poor) sanitation status have no latrine or toilet facility. Households with improved (good) sanitation status have a pour-flush latrine, or ventilated improved pit latrine. Poor hygiene practice includes having no hand-washing and bathing facilities or detergents in the house, or washing hands with water but no soap or other detergents. Good hygiene practices include the use of hand-washing and bathing facilities, with the availability of soap and other detergents in the house.

### Variables

Three types of variables were considered in the study: sociodemographic (age, sex, occupation, ethnicity, religion, type of housing); environmental (drinking water sources, presence or absence of latrines at home, wastewater management and domestic waste management, hygiene status), and the prevalence of BU, obtained from the register of the Centre de Dépistage et de Traitement de l'Ulcère de Buruli in Lalo (CDTUB LALO).

### Data processing and analysis

The data were checked, coded, and entered in Excel and analyzed using Statistical Package for Social Science (SPSS) version 19.0. Univariate analysis was conducted. Using logistic regression, multivariate analysis was also carried out. The odds ratio, and 95 % confidence interval (CI) were used to determine the effect of potential associated variables on the sanitation and hygiene status considered as outcomes variables and to control confounding factors. The transcripts of the qualitative data were coded using a coding scheme and analyzed across selected themes and triangulated with data from the questionnaire.

### Ethical considerations

Ethical clearance was obtained from the National Ethical Review Board of the Ministry of

Health Benin (N°147/MS/DC/SGM/DFRS/CNPERS/SA). The questions from the questionnaire were proven not to affect the morale or personality of study subjects. Written informed consent was obtained from each study subject after they had been given an explanation of the research, and what they were required to do and told that their involvement was voluntary. Confidentiality was assured by using code numbers rather than names and keeping questionnaires locked up. Data collectors also gave health education and advice to the subjects during the data collection process.

## Results

### Demographic characteristics of respondents

The Table 1 shows the sociodemographic and environmental characteristics of the households. The majorities were farmers and more than 73 % of household heads had received no formal education. Only 7.67 % (46) of households lived in houses built with sustainable materials. The majority of households lived in houses built with flimsy materials, reflecting their economic poverty.

**Table 1:** Demographic characteristics of households

	Variables	Frequency	Percent %	95 % CI
Sex	Male	455	75.80 %	[72.2–79.2]
	Female	145	24.20 %	[20.8–27.8]
Total		600	100	
Occupation	Seller	20	3.33	[2.10–5.19]
	Farmer	545	90.83	[88.1–92.9]
	Craftsman	15	2.5	[1.5–4.2]
	Teacher	10	1.67	[0.85–3.15]
	Other	10	1.67	[0.85–3.15]
Total		600	100	
Ethnicity	Adja	363	60.5	[56.4–64.4]
	Fon	209	34.8	[31–38.8]
	Other	28	4.7	[3.2–6.8]
Total		600	100	
Education	Illiterate	439	73.17	[69.40–76.64]
	Able to read and write	161	26.83	[23.36–30.60]
Total		600	100	
Type of housing	Using modern building materials	46	7.67	[5.72–10.17]
	Using flimsy building materials	554	92.33	[89.83–94.28]
Total		600	100	

### Environmental characteristics of household

The Table 2 shows the environmental characteristics of the households. More than 49 % of households used unimproved sources of water on a daily basis. Only 8.67 % (62) households had improved sanitation facilities at home and 9.7 % (58) households had improved hygiene behavior. 16 % (96) had permanent availability of soap at home.

**Table 2:** Environmental characteristics of households

	Variables	Frequency	Percent %	95 % CI
Water sources	Piped water (improved)	280	46.67	[42.63–50.75]
	Wells	24	4	[2.64–5.98]
	Groundwater	296	49.33	[45.27–53.41]
Total		600	100	
Water status	Improved	280	46.67	[42.6–50.8]
	Unimproved	320	53.3	[4902–57.8]
Total		600	100	
Sanitation	Improved	52	8.67	[6.60–11.28]
	Unimproved	548	91.33	[88.7–93.4]
Total		600	100	
Availability of soap	Yes	96	16.00 %	[13.2–19.20]
	No	504	84.00 %	[80.8–86.8]
Total		600	100	
Handwashing practices	No hand washing	419	69.8	[66.0–73.5]
	With water only	123	20.5	[17.4–24.0]
	With water and soap	58	9.7	[7.5–12.4]
Total		600	100	
Hygiene status	Unimproved	542	90.3	[87.6–92.5]
	Improved	58	9.7	[7.5–12.4]
Total		600	100	

### Cumulative number of cases of BU in the district of Lalo from 2006 to 2012

The Table 3 shows the number of cases of BU detected and treated at the CDTUB LALO from 2006 to 2012. BU appears to be a common condition in this commune with a relatively high detection rate. The sub-districts with the highest levels were Adoukandji, Gnizounmè, Ahomadegbé, and Tchito with 253, 192, 135 and 100 current BU cases respectively. Lokogba, Banigbé and Zalli had lower levels.

**Table 3:** Cumulative detection of BU in subdistricts of Lalo

Subdistrict	Cumulative new cases detected (2006–2012)	Mean population (2006–2012)	Cumulative detection Rate
Ahomadégbé	135	4565	30/1000
Adoukandji	253	11035	23/1000
Gnizounmè	192	9571	20/1000
Tchito	100	6155	16/1000
Ahodjinnako	79	6279	13/1000
Tohou	45	6749	07/1000
Hlassamey	72	16924	04/1000
Banigbé	8	6454	01/1000
Lalo Centre	13	14583	01/1000
Lokogba	8	10091	01/1000
Zalli	12	36493	00/1000
Total lalo	917	102298	09/1000

### Factors associated with sanitation and hygiene status in the district of Lalo

The operational definitions of improved sanitation, good hygiene practices were based on the WHO/UNICEF 2013 report on WASH [8], as describe in the method section.

Based on theses operational definitions, the Tables 4 and 5 show the results of the multivariate analysis of factors associated with sanitation. The type of housing as an indicator of socioeconomic status, the permanent availability of soap and improved hygiene status were identified as the main factors positively associated with improved sanitation and hygiene.

**Table 4:** Factors associated with hygiene and sanitation status

		Unimproved	Improved	Crude OR	95 % CI	Adjusted OR	95 % CI	P
Education	Illiterate	412	27	2.81	1.57–5.00	1.49	0.75–2.94	0.25
	Read and write	136	25	1.0		1.0		
Type of housing	Modern materials	31	15	0.15	0.07–0.30	0.32	0.14–0.75	0.01
	Flimsy materials	517	37	1.0		1.0		
Availability of soap	Yes	64	32	0.08	0.04–0.15	0.17	0.08–0.34	0.00
	No	484	20	1.0		1.0		
Hygiene status	Improved	37	21	0.11	0.06–0.20	0.32	0.18–0.69	0.00
	Unimproved	511	31	1.0		1.0		
Diarrhea in the last 7 days	No	296	42	0.28	0.14–0.57	0.48	0.22–1.02	0.06
	Yes	252	10	1.0		1.0		

Note: Underlining indicates significance at the 0.05 level

**Table 5:** Factors associated with hygiene status

		Unimproved	Improved	Crude OR	95 % CI	Adjusted OR	95 % CI	P
Education	illetrate	409	30	2.87	1.65–4.98	1.84	0.98–3.43	0.06
	Read and write	133	28	1.0		1.0		
Type of housing	Modern materials	32	14	0.20	0.10–0.40	0.48	0.20–1.11	0.09
	Flimsy materials	510	44	1.0		1.0		
Availability of soap	Yes	63	33	0.10	0.056–0.18	0.19	0.10–0.36	0;00
	No	479	25	1.0		1.0		
Sanitation	Unimproved	511	37	9.36	4.90–17.86	3.23	1.51–6.91	0.00
	Improved	31	21	1		1.0		
Diarrhea in the last 7 days	No	294	44	0.38	0.20–0.71	0.70	0.35–1.40	0.31
	Yes	248	14	1				

Note: Underlining indicates significance at the 0.05 level

### Results of in-depth interviews

In-depth interviews with key informants were used to gain a better appreciation of their basic knowledge of hygiene and sanitation.

### Water uses

Consumption of surface water was recognized by the community as having a health risk, with several making comments like: “The water contains microbes and gives us diseases”. The lack of improved water sources forces the households to use this water. It is paradoxical to note that the population generally does not use any disinfection measures, saying, for example: “We have always drunk water like that” or “We do not have disinfectants”. Transporting drinking water in non-covered basins was common because “We’ve always done it like that”. The risk associated with the disposal of leaves and plastic bags in drinking water is well known, with comments being made such as: “It’s because there was dirt on the leaves that we got sick”.

#### Sanitation

Open defecation is most frequently used by the majority (91, 32 %) of the households. This is explained by the absence of latrines, probably because of the low economic level of most households. The resulting risks are well-known and people are aware that the construction of latrines would be helpful to address the problem. The in-depth interviews did not reveal any cultural barriers to the use of latrines. However a reticence was noted about the use of community latrines, due to infrastructure maintenance problems.

#### Waste management

Waste is typically discharged into the surrounding environment by almost all of the household. Some of the households incinerate waste periodically. The almost total absence of controlled garbage dumps and the lack of garbage collection systems reflect the lack of attention to this problem, and waste management is a major concern in the municipality.

#### Hand-washing practices

Washing hands before eating is a common practice. However, it is often not practical for some meals and may be omitted in certain circumstances (for example, on the farm). This washing, however, is not always with soap because of the cost. Hand-washing with soap after defecation is practiced by a little part of households. The non-availability of water influences hand washing, with comments including: “We already have insufficient water for drinking purposes, so it’s hard to wash our hands with the little water we have”.

According to our results, the minimum requirements of good hygiene and good sanitation are not met. There is a lack of garbage dumps and waste management infrastructure, an almost total lack of latrines in households, and low access to improved water sources.

## Discussion

Numerous aspects of the control of NTDs require individuals to have good access to WASH services. This may include water to practice good skin hygiene as well as access to good sanitation for those affected [1]. As far as Buruli ulcer is concerned, contrary to the WASH preventable NTDs [2], there are currently few studies on the WASH and BU. A study conducted in Cameroon show persistence of *M. ulcerans* specific DNA sequences over a period of more than two years at a water contact location of BU patients in an endemic village of Cameroon. At defined positions in a shallow water hole used by the villagers for washing and bathing, detritus remained consistently positive for *M. ulcerans* DNA. The result of real-time PCR indicated *M. ulcerans*, which cause human disease, persisted in this environment after successful treatment of all local patients. Underwater decaying organic matter may therefore represent a reservoir of *M. ulcerans* for direct infection of skin lesions or vector-associated transmission [9]. In the same line, in a study conducted in Benin, a total of 416 participants were enrolled including 104 cases and 312 controls. BU history in the family (p < 0.001), adjusted by daily contact with a natural water source (p < 0.007), was significantly associated with higher odds of having BU (OR; 95 % CI = 5.5; 3.0–10.0) [10]. In addition to these considerations, as we explain in the background of our study, the WASH sector is vital for the management of BU patients need water for wound care, scar management after skin grafts, taking medications, and for good personal hygiene to prevent complications and secondary infections. Washing the skin lesions with clean water and soap could be protective for those exposed to BU [6]. Studies on wound care (an important component of the management of BU) have shown that washing wounds daily with clean water and soap is effective in reducing the risk of contamination by various microorganisms [7]. In the fight against HIV/AIDS, good access to WASH services can prevent opportunistic infections and improve patients’ lives [11, 12]. Adequate access to WASH services is therefore a challenge for both healthy and non-healthy populations in many countries and communities with high levels of NTDs [1].

This first study in a district in Benin where BU is endemic is very timely, and provides an inventory of WASH indicators in the study area. The average (mean) age of household heads interviewed was 45 years, with a mode of 35 and a standard deviation of 10.42. Heads of households were mostly farmers and their ethnicity is Adja (60.50 %). Other ethnicities (7 %) identified were Yoruba, Fon, Mina and some Bariba. These data are consistent with figures from the municipality showing that the population of the town is dominated by the Adja ethnic group, which together with Fon makes up 95.3 % of the population [13].

The population of Lalo uses three sources of water for drinking purposes: borehole (46.67 %), rainwater tank (4 %); and surface water (49.33 %). Generally, people had experience of using disinfection techniques such as chlorination, but did not implement them, for various reasons, probably the extra work or additional costs required This cross-sectional study was not able to assess the functionality of boreholes throughout the year, nor did it investigate the quality of water from them. However, in many poor countries, studies have shown that the water supplied by the water distribution systems may be poor quality [8]. A study conducted by our team in another municipality of Benin corroborates these findings [14]. Similarly, a study carried out in the municipality of Abomey Calavi in Benin demonstrated that the water from the aquifer was contaminated by various microorganisms including *Escherichia coli*, *Klebsiella pneumoniae*, *Staphylococcus aureus*, *Salmonella spp*, *Clostridium perfringens* and fecal streptococci [15]. In some localities in Lalo, such as the villages of Djibahoun and Assogbahoué, there is no system for supplying improved water for the population. Households there use only unimproved water sources. The results from this study therefore show that 53.33 % of households use unimproved water (surface water and rainwater tank) for drinking purposes. This proportion is higher than that in the WHO and UNICEF report published in 2013 [8] on Benin, which suggested that about 43 % of rural households had access to improved water sources.

There was low latrine coverage for households: only 8.67 % of those surveyed. This low rate is in line with official data from Lalo, which reveal that there are almost no household latrines in the majority of villages in the commune [13]. A study by Reiff et al. for the Global Sanitation Fund and Water Supply and Sanitation Collaborative Council established an evacuation of excreta rate of about 30.21 % in the Atlantique region [16].

As well as the questionnaire survey, which revealed a very low rate of hand-washing, we also made a count of latrines equipped with hand-washing facilities, to obtain an indirect estimate of the frequency of this practice. We found no latrines with hand-washing devices. This exposes people to the risk of disease, because the lack of hand-washing facilitates the transmission of fecal diseases. For example, Judah et al. reported that 28 % of frequent travelers had fecal bacteria on their hands [17].

Observation of maternal practices in handling children’s feces shows that often, no precautions are taken. This situation encourages the transmission of fecal diseases. Gil et al. [18] showed that improper disposal and unsanitary handling practices of children’s feces were associated with an increase of 23 % in the risk of diarrhea, which may encourage people to increase hand-washing [19, 20]. Despite this, our study showed that systematic hand-washing after defecation remains marginal. This is not without consequences for health, as our field survey found that children had suffered from diarrhea in 11.67 % of households. This could be explained by the behavior of children, who defecate on the floor and frequent uncontrolled garbage dumps. Studies in other African countries have shown a strong link between exposure to solid waste released into the environment and diarrheal diseases. For example, Dikassa et al. [21] demonstrated that in Kinshasa, the children of families living in very poor hygienic and sanitary conditions were at 70 % greater risk of suffering from severe diarrhea.

Wastewater is poorly managed in the district of Lalo. All the households surveyed lacked home cesspools, which are recommended by the Ministry of Health [22]. Wastewater, as well as solid waste, is released untreated by 98.17 % of households. These practices pose a risk to public health. In some villages, households use garbage as fertilizer on family plantations. These waste management methods should be encouraged in rural areas.

Several factors can influence the sanitation status such as level of education, type of housing, hygiene practices, and water availability [8]. In our multivariate model, the type of housing as an indicator of socioeconomic status was identified as the main factor positively associated with improved sanitation. The lack of soap and the absence of hand-washing were the main factors associated with low sanitation level. We found no link between sanitation and the level of education of the household head, in contrast to a study conducted in Ethiopia, which showed that children’s hygiene practices at school were dependent on the parents’ level of education [23]. The same observation was made by Schmidt et al. in Kenya, who showed that hand-washing practices were dependent on level of education [24].

Measured as the proportion of people living on less than $ 1 a day, half of the departments in Benin are severely affected by poverty, including Couffo, where this study was conducted. There, the proportion of the population affected by this extreme poverty is estimated to be between 61 % and 75 % [25]. Our results are consistent with those of the Water and Sanitation Program, which also showed a disparity between rich and poor in terms of access to sanitation in Benin [26]. It is therefore clear that the municipality of Lalo lacks individual and collective sanitation, has a shortage of improved water supply infrastructure and poor hygiene practices.

It would be helpful to plan and implement targeted interventions to correct this situation. UNICEF and WHO recommended a strategy called community-led total sanitation, to gradually move villages to zero open defecation [27]. This strategy requires strong community mobilization, but our results suggest that it would be relevant in Lalo. Community programs for early detection of Buruli ulcer and other neglected tropical diseases of the skin could benefit from this social mobilization. Wound care and management of scars after skin grafts can also be taken into account in these WASH programs.

## Conclusion

In the commune of Lalo, one of the four districts in Benin where levels of BU are highest, water hygiene and sanitation levels indicators are very low. This has obvious effects on health, especially for children. This study provides baseline information for future interventions in the WASH sector in this municipality. Those treating BU and other NTDs can use the WASH platform to share resources to make interventions more efficient for communities, partners and the health system.

## Abbreviations

NTDs: Neglected tropical diseases
WASH: Water sanitation and hygiene
BU: Buruli ulcer
